# A comprehensive report of disease complications of juvenile idiopathic arthritis using a prospective cohort study

**DOI:** 10.1186/s12969-025-01180-6

**Published:** 2025-12-22

**Authors:** Jacob Anderson, Kaleo Ede, Nikita Goswami, Michael Shishov, Geoffrey Thiele, Vinay Vaidya, Elisa Wershba

**Affiliations:** 1 Department of Pediatric Rheumatology, Phoenix Children’s Hospital, Phoenix, AZ USA; 2 Department of Pediatric Rheumatology, University of Utah, 81 North Mario Capecchi Drive, Rm 4a003, Salt Lake City, UT 84113 USA

**Keywords:** Juvenile idiopathic arthritis, Disease complications, Joint damage, Uveitis, Growth failure, Biologics

## Abstract

**Background:**

Given the chronic inflammatory nature of juvenile idiopathic arthritis (JIA), disease complications may occur including chronic joint damage, ocular complications, and growth restrictions. Few studies have provided a comprehensive assessment of disease complication rates in a contemporary population of patients with JIA. The primary objective of this study was to provide a comprehensive report of disease complications in our JIA population. Secondary objectives included characterizing patients who developed complications and identifying associated risk factors.

**Methods:**

We conducted a prospective cohort study. A standardized list of JIA disease complications was created by consensus among rheumatologists and included in the electronic medical record. A total of 432 patients diagnosed with JIA for more than one year were evaluated at our rheumatology clinic between January 2023 and February 2024 and were assessed for the development of disease complications. Univariate analysis was used to test for associations between the presence of complications and potential risk factors such as sex, age of disease onset, diagnosis duration, serology positivity, and treatment history.

**Results:**

Of the 432 patients, 146 (33.8%) were identified to have at least one disease complication. Joint complications occurred in 73 patients (16.9%), and uveitis was identified in 57 patients (13.2%), with 14 (25%) of those experiencing secondary ocular complications. Growth failure was observed in 4% of patients, with short stature resulting in 1%. Younger age at disease onset and longer time to initiation of biologic therapy were associated with an increased risk of developing complications. Additionally, delayed initial rheumatology evaluation and delayed initiation of biologic therapy were both associated with a progressively increasing risk of joint complications.

**Conclusion:**

Despite therapeutic advances in the treatment of JIA, a substantial proportion of patients continue to develop disease complications. Approximately one-third of patients in our cohort experienced at least one disease complication. Delayed initiation of biologic therapy was associated with higher complication rates, highlighting the importance of early therapeutic intervention to reduce the burden of disease complications.

## Background

Juvenile Idiopathic Arthritis (JIA) is the most common rheumatologic disease in childhood, affecting approximately 300,000 children in North America [1]. The seven subtypes of JIA include systemic JIA, oligoarticular, rheumatoid factor (RF) positive polyarticular, RF negative polyarticular, psoriatic arthritis, enthesitis-related arthritis (ERA), and undifferentiated arthritis [2].

JIA is a chronic disease that may persist into adulthood. While some patients achieve disease remission, others experience persistent or recurrent symptoms. Given the chronic inflammatory nature of JIA, disease complications may occur including chronic joint damage, ocular complications, and growth restrictions [3].

Chronic inflammation associated with JIA may result in joint-related complications such as chronic arthropathies, joint deformities, micrognathia, limb length discrepancies, and cervical instability [3]. Uveitis affects approximately 10–20% of patients with JIA, with increased risk observed in children with oligoarticular JIA, those diagnosed at age ≤ 7 years, and those with positive antinuclear antibodies (ANA) [4, 5]. Complications of JIA-associated uveitis include cataracts, posterior synechiae, cystoid macular edema, glaucoma, and band keratopathy [6]. Additionally, chronic disease activity can impair linear growth, and can result in short stature, with final height z-score < -2.0 [7]. Macrophage activation syndrome (MAS), a life-threatening complication seen in systemic JIA, occurs in approximately 10% of affected individuals [8].

Although specific complications of JIA have been studied, few investigations have provided a comprehensive assessment of complication rates across a broad JIA population. Two studies from India have described such complications. In 2017, Dewoolkar et al. reported on complications among patients with systemic JIA, including joint deformities, limb length discrepancy, micrognathia, MAS, infections, osteoporosis, avascular necrosis of the femoral head, and cataracts [9]. In 2018, Menon et al. described complications in a population of JIA patients including MAS, articular damage, limb length discrepancy, growth failure, pubertal delay, osteoporosis, avascular necrosis, abnormal vertebral curve, uveitis, cataracts, subcutaneous atrophy, striae rubrae, and amyloidosis [10]. To our knowledge, no study has provided a comprehensive assessment of complications in a population of JIA patients in the United States.

Furthermore, most studies assessing disease complications in JIA have been retrospective. To our knowledge, no study to date has been conducted using a prospective study design to provide a comprehensive report of disease complications. As the accuracy of retrospective studies may be disadvantaged due to missing information and lack of standardized definitions or documentation, more prospective studies are needed to more accurately identify rates of disease complications [11].

The primary objective of this study is to provide a comprehensive report of disease complications in our JIA population using a prospective cohort study design. Specifically, we aim to determine the prevalence of disease complications including chronic arthropathies, joint deformities, micrognathia, limb length discrepancies, cervical instability, MAS, growth failure, uveitis, and secondary ocular complications. Secondary objectives of this study are to (1) characterize demographics of patients with JIA who develop complications and (2) Assess for predictive factors associated with the development of complications including age of disease onset, sex, serology positivity (ANA, RF, cyclic citrullinated peptide (CCP), human leukocyte antigen B27 (HLA-B27)), and treatment history.

Understanding the prevalence of complications and identifying predictive risk factors are essential for recognizing which patients will develop these complications. This knowledge is critical to guide the early use of targeted therapies aimed to prevent long-term complications.

## Methods

### Study design and population

This study was a prospective cohort study to evaluate disease complications in a population of patients with JIA using both quantitative and qualitative analysis. Approval to conduct this study was obtained by Phoenix Children’s Institutional Review Board (IRB-24-174). The primary objective of this study is to provide a comprehensive report of disease complications in our JIA population. Specifically, we aim to determine the prevalence of disease complications including chronic arthropathies, joint deformities, micrognathia, limb length discrepancies, cervical instability, MAS, growth failure, uveitis, and secondary ocular complications (cataracts, posterior synechiae, cystoid macular edema, glaucoma, and band keratopathy). Secondary objectives of this study are to (1) characterize demographics of patients with JIA who develop complications and (2) Assess for predictive factors associated with the development of complications including age of disease onset, sex, serology positivity (ANA, RF, CCP, HLA-B27), and treatment history. A standardized list of JIA disease complications was created by consensus among rheumatologists and included in the electronic medical record. A total of 483 patients diagnosed with JIA for more than one year and seen at our rheumatology clinic between January 2023 and February 2024 were screened. Inclusion criteria included: arthritis onset before the age of 16 years; documentation of the presence or absence of complications completed at the most recent visit during the study period. All JIA subtypes and demographic groups were included. JIA subtypes were classified according to the International League of Associations for Rheumatology (ILAR) criteria [2]. The percentage of patients with documented complication data was tracked and reviewed at weekly division meetings.

After the study period, a chart review was performed for patients with completed complication documentation. A total of 432 patients met inclusion criteria and were included in the analysis.

Disease and treatment complications were categorized as “present” or “absent”. Disease activity was assessed using active joint count (AJC) and clinical juvenile arthritis disease activity score (cJADAS) as recorded in the clinical documentation. Height was evaluated using the World Health Organization (WHO) growth charts for ages 0–2 years, and Centers for Disease Control and Prevention (CDC) growth charts for ages 2–20 years [12–13]. Growth failure was defined as a decrease in height z-score > 1.0 and short stature as a final z-score < -2.0. Patients with underlying genetic conditions known to affect height were excluded from short stature assessment.

### Statistical analysis

Data were summarized using frequencies and proportions for categorical variables and mean and standard deviation for continuous variables and 95% confidence intervals (95% CI) for proportions of categorical variables of interest were calculated. Univariate analysis was used to test for associations between risk factors (e.g., JIA subtype, sex, age at symptom onset, age at first rheumatology clinic visit, disease duration, serology positivity, time until methotrexate (MTX) initiation, time until biologic initiation, number of biologics and/or Janus kinase inhibitors (JAKi) failed) and current disease activity with the primary outcome. Relative risks (RR) and 95% CIs were reported. A *p*-value of < 0.05 was considered statistically significant. Statistical analyses were performed using the statistical software package SPSS and Microsoft Excel.

The large language model, OpenEvidence, was used minimally to assist with researching of literature. However, the majority of literature review was performed without the aid of large language models. No other aspects of this study were conducted with the assistance of large language models.

## Results

### Study population characteristics

A total of 483 patients diagnosed with JIA for more than one year were seen in our clinic during the study period. Documentation of complications were completed for 89.4% of patients, with a total of 432 patients meeting the inclusion criteria for this study. Mean age of disease onset for our JIA cohort was 6.9 ± 4.9 years, with a mean disease duration of 6.2 ± 4.3 years. Of the 432 patients, 68.3% (295) were female. Classification of JIA subtypes were as follows: 8.1% (35/432) had systemic JIA, 25.5% (110/432) had RF negative polyarticular, 10.2% (44/432) had RF positive polyarticular, 28.9% (125/432) had persistent oligoarticular, 2.3% (10/432) had extended oligoarticular, 12.5% (54/432) had ERA, 3% (13/432) had psoriatic arthritis, and 9.5% (41/432) had undifferentiated JIA. Among patients tested, 14% (56/399) were positive for RF, 15.3% (56/367) for anti-CCP antibodies, 24.9% (62/249) for HLA-B27, and 24.9% (103/414) for ANA (Table 1).

**Table 1:** Population demographics

	*N* = 432
Age of onset, yrs, mean ± SD	6.9 ± 4.9
Diagnosis duration, yrs, mean ± SD	6.2 ± 4.3
Female sex	295 (68.3%)
Subtype	
Systemic	35 (8.1%)
RF negative polyarticular	110 (25.5%)
RF positive polyarticular	44 (10.2%)
Oligo-persistent	125 (28.9%)
Oligo-extended	10 (2.3%)
ERA	54 (12.5%)
Psoriatic	13 (3%)
Undifferentiated	41 (9.5%)
Serologies*	
RF positive	56 / 399 (14%)
Anti-CCP positive	56 / 367 (15.3%)
HLA-B27 positive	62 / 249 (24.9%)
ANA positive	103 / 414 (24.9%)

Data shown as n (%) unless otherwise specified

*Frequencies for serology positivity shown as number of patients positive over total patients tested. ANA: anti-nuclear antibody; CCP: citric citrullinated peptide; ERA: enthesitis-related arthritis; RF: rheumatoid factor

At the time of the most recent clinic visit, 81.9% (354/432) had an AJC of zero, while 18.1% (78/432) of patients had an AJC > 0. A cJADAS score was assessed for 425 patients. The mean cJADAS score for all patients was 2.76 ± 4.56, with 48% (206/425) patients having a cJADAS score of zero, indicating inactive disease.

Assessment of treatment history revealed that 31% (134/432) patients were not on any current treatment regimen at time of last visit. Conversely, 4.6% (20/432) were being treated with nonsteroidal anti-inflammatory drugs (NSAIDS) alone, MTX or other disease-modifying antirheumatic drugs (DMARD) alone for 8.8% (38/432) of patients, tumor necrosis factor inhibitors (TNFi) alone for 32.2% (139/432) of patients, a combination of DMARD + TNFi for 7.4% (32/432) of patients, other biologics or JAKi alone for 14.4% (62/432) of patients, and a combination of DMARD + other biologic or JAKi for 1.6% (7/432) of patients. Additionally, 73.2% (312/426) of patients had a history of monotherapy treatment with MTX, with 76.3% (238/312) of these patients failing MTX monotherapy. Overall, 67.6% (292/432) of patients had a history of treatment with a TNFi or other biologic therapy.

### Disease complications

Overall, 33.8% (146/432) of patients experienced at least one disease complication. Joint-related complications were observed in 16.9% (73/432) of patients. Chronic arthropathy occurred in 10.4% (45/432) of patients, joint deformities in 3.9% (17/432), leg length discrepancies in 3.9% (17/432), and 0.9% (4/432) developed micrognathia. No patients were found to have cervical instability. The most common joints found to have chronic changes were the joints of the fingers (*n* = 25), followed by wrists (*n* = 16) (Table 2).

Uveitis was identified in 13.2% (57/432) of patients. Of the 57 patients with uveitis, 25% (14/57) experienced secondary ocular complications. These included cataracts in 21% (12/57), glaucoma in 14% (8/57), posterior synechia in 9% (5/57), and band keratopathy in 5% (3/57). No patients were found to have developed cystoid macular edema. Only one patient with uveitis also developed a joint complication (Table 2).

Growth failure was identified in 3.8% (15/432) of patients. Of patients with growth failure, 5 patients had systemic JIA, 4 had persistent oligoarticular, 2 had extended oligoarticular, 3 had ERA, and 1 had undifferentiated JIA. Of those with growth failure, 9 patients were female and 6 were male. Of these patients, 5 patients had resultant short stature. All patients with short stature were female (Table 2).

**Table 2:** Rates of disease complications by JIA subtype

	All	Systemic	RF negative polyarticular	RF positive polyarticular	Oligo- persistent	Oligo- extended	ERA	Psoriatic	Undifferentiated
	*N* = 432	*n* = 35	*n* = 110	*n* = 44	*n* = 125	*n* = 10	*n* = 54	*n* = 13	*n* = 41
Any complication	146 (34%)	13 (37.1%)	42 (38.2%)	8 (18.2%)	51 (40.8%)	4 (40%)	9 (16.7%)	4 (30.8%)	15 (36.6%)
Joint complications									
Any joint complication	73 (16.9%)	3 (8.6%)	25 (22.7%)	7 (15.9%)	19 (15.2%)	2 (20%)	4 (7.4%)	2 (15.4%)	11 (26.8%)
Chronic arthropathy	45 (10.4%)	2 (5.7%)	17 (15.5%)	5 (11.4%)	11 (8.8%)	0 (0%)	3 (5.6%)	1 (7.7%)	6 (14.6%)
Joint deformity	17 (3.9%)	0 (0%)	7 (6.4%)	2 (4.5%)	1 (0.8%)	1 (10%)	1 (1.9%)	0 (0%)	5 (12.2%)
Micrognathia	4 (0.9%)	0 (0%)	3 (2.7%)	0 (0%)	1 (0.8%)	0 (0%)	0 (0%)	0 (0%)	0 (0%)
Limb length discrepancy	17 (3.9%)	1 (2.9%)	2 (1.8%)	0 (0%)	9 (7.2%)	1 (10%)	0 (0%)	1 (8%)	3 (7.3%)
Cervical instability	0 (0%)	0 (0%)	0 (0%)	0 (0%)	0 (0%)	0 (0%)	0 (0%)	0 (0%)	0 (0%)
Ocular complications									
Uveitis	57 (13.2%)	0 (0%)	17 (15.5%)	1 (2.3%)	31 (24.8%)	0 (0%)	3 (5.6%)	2 (15.4%)	3 (7.3%)
Any secondary ocular complication*	14 (24.5%)	0 (0%)	5 (29.4%)	0 (0%)	8 (25.8%)	0 (0%)	0 (0%)	0 (0%)	1 (33.3%)
Cataract*	12 (21.1%)	0 (0%)	3 (17.6%)	0 (0%)	8 (25.8%)	0 (0%)	0 (0%)	0 (0%)	1 (33.3%)
Posterior synechiae*	5 (8.8%)	0 (0%)	1 (5.9%)	0 (0%)	3 (9.7%)	0 (0%)	0 (0%)	0 (0%)	1 (33.3%)
Cystoid macular edema*	0 (0%)	0 (0%)	0 (0%)	0 (0%)	0 (0%)	0 (0%)	0 (0%)	0 (0%)	0 (0%)
Glaucoma*	8 (14%)	0 (0%)	1 (5.9%)	0 (0%)	7 (22.6%)	0 (0%)	0 (0%)	0 (0%)	0 (0%)
Band keratopathy*	3 (5.3%)	0 (0%)	0 (0%)	0 (0%)	2 (6.5%)	0 (0%)	0 (0%)	0 (0%)	1 (33.3%)
Growth complications									
Growth failure	15 (3.5%)	5 (14.3%)	0 (0%)	0 (0%)	4 (3.2%)	2 (20%)	3 (5.6%)	0 (0%)	1 (2.4%)
Short stature	5 (1.2%)	3 (8.6%)	0 (0%)	0 (0%)	1 (0.8%)	0 (0%)	0 (0%)	0 (0%)	1 (2.4%)
Other complications									
MAS	-	7 (20%)	-	-	-	-	-	-	-

Data shown as n (%). *Secondary uveitis complications show proportion with uveitis who developed the complication. Growth failure is defined as a decrease in height z-score > 1.0. Short stature as a final z-score < -2.0. ERA: Enthesitis-related arthritis; MAS: Macrophage activation syndrome; RF: Rheumatoid factor

### Characterizing patients with disease complications

Patients with disease complications had an earlier mean age of disease onset compared to those without complications (5.3 ± 3.9 vs. 7.7 ± 4.8 years, *p* < 0.001), and a longer mean disease duration (7.6 ± 4.6 vs. 5.4 ± 3.8 years, *p* < 0.001). These differences were predominantly found in patients with uveitis; no significant differences in disease onset or diagnosis duration were found between patients with and without joint complications.

No significant difference was observed in time from disease onset to the first rheumatology visit for patients with any disease complication (0.6 ± 1.2 vs. 0.5 ± 1.0 years, *p* = 0.56). However, patients with joint complications experienced a longer time to their first rheumatology visit (0.9 ± 1.5 vs. 0.5 ± 1.0 years, *p* = 0.003), whereas patients with uveitis had a shorter time to their first visit (0.3 ± 0.4 vs. 0.6 ± 1.1 years, *p* = 0.046).

Patients with any disease complication had a significantly longer time from initial rheumatology visit to the initiation of methotrexate (1.0 ± 2.0 vs. 0.4 ± 0.9 years, *p* < 0.001) and to the initiation of their first biologic treatment (2.2 ± 2.9 vs. 0.9 ± 1.2 years, *p* < 0.001). These delays were isolated to patients with uveitis who were found to have a longer time to starting methotrexate (1.7 ± 2.8 vs. 0.4 ± 0.8 years, *p* < 0.001) and to starting biologic treatment (3.5 ± 4.1 vs. 1.0 ± 1.6 years, *p* < 0.001), while no differences were seen in patients with joint complications.

There was no significant difference in the number of biologic and/or JAKi therapies failed among patients with any disease complications overall (1.0 ± 1.5 vs. 0.9 ± 1.2, *p* = 0.39). However, patients with joint complications failed a greater number of biologics and/or JAKi compared to those without joint complications (1.4 ± 2.0 vs. 0.8 ± 1.2, *p* = 0.004) (Table 3).

**Table 3:** Characterizing patients with disease complications

	All complications			Joint complications			Uveitis		
	No complication *n* = 286	Any complication *n* = 146	*P*	No joint complication *n* = 359	Joint complication *n* = 73	*P*	No Uveitis *n* = 375	Uveitis *n* = 57	*P*
Age of onset, yrs	7.7 ± 4.8	5.3 ± 3.9	**< 0.001**	7.1 ± 4.7	6.2 ± 4.2	0.14	7.3 ± 4.7	3.8 ± 2.6	**< 0.001**
Time until 1st visit, yrs	0.5 ± 1.0	0.6 ± 1.2	0.56	0.5 ± 1.0	0.9 ± 1.5	**0.003**	0.6 ± 1.1	0.3 ± 0.4	**0.046**
Diagnosis duration, yrs	5.4 ± 3.8	7.6 ± 4.6	**< 0.001**	6.1 ± 4.3	6.1 ± 3.9	0.98	5.6 ± 3.9	9.5 ± 4.9	**< 0.001**
Time to methotrexate, yrs	0.4 ± 0.9	1.0 ± 2.0	**< 0.001**	0.7 ± 1.6	0.4 ± 0.6	0.26	0.4 ± 0.8	1.7 ± 2.8	**< 0.001**
Time to biologic, yrs	0.9 ± 1.6	2.2 ± 2.9	**< 0.001**	1.3 ± 2.4	1.7 ± 1.7	0.25	1.0 ± 1.6	3.5 ± 4.1	**< 0.001**
Number of biologics/JAKi failed	0.9 ± 1.2	1.0 ± 1.5	0.39	0.8 ± 1.2	1.4 ± 2.0	**0.004**	1.0 ± 1.4	0.6 ± 1.1	0.08

Data shown as mean ± SD unless otherwise specified

Patients with uveitis who developed secondary ocular complications had a longer disease duration than those without secondary ocular complications (12.0 ± 3.7 vs. 8.7 ± 5.0 years, *p* = 0.03). No other significant differences were identified between these groups.

### Assessing risk factors of developing disease complications

**Fig. 1:**
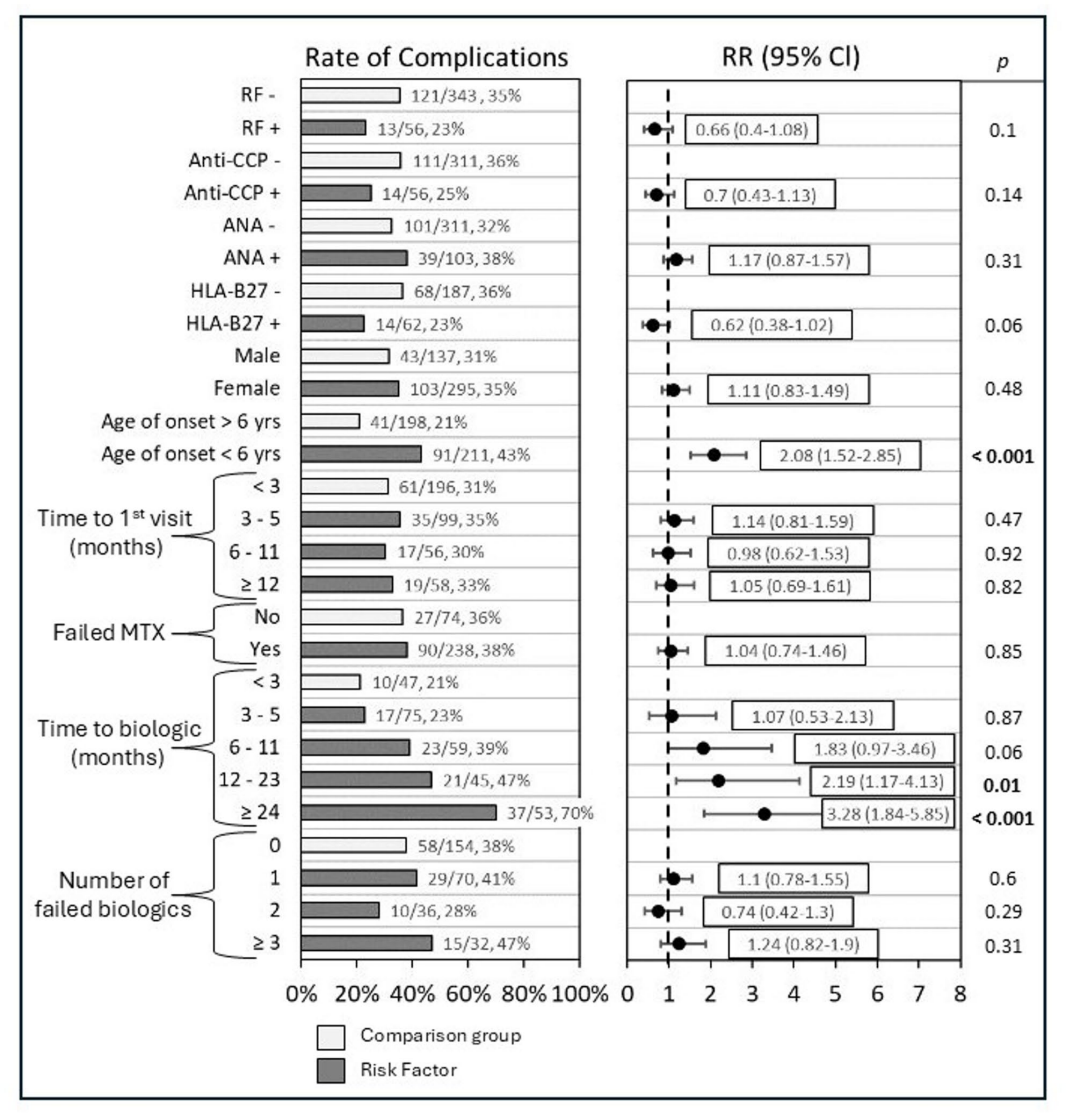
Risk Factors of developing any disease complication. Shows proportion of patients of various factors who developed any disease complication. Relative risk with 95% confidence internal shown. Light bars represent comparison group, while dark bars show risk factors assessed for relative risk with 95% confidence internal as compared to the comparison group

Factors found to have statistically significant relative risk of developing a disease complication included young age of disease onset and increased time to initiating biologic therapies. Among patients with disease onset before age 6 years, 43.1% (91/211) developed a disease complication, compared to 21% (41/198) of patients with disease onset at age 6 years or older (RR 2.08, CI 1.52–2.85, *p* < 0.001). Time to biologic therapy initiation was associated with increased relative risk to developing a disease complication. Specifically, 21% (10/47) of patients who began biologic therapy within 3 months of their first rheumatology visit developed a disease complication, compared to 23% (17/75) of patients starting between 3 and 5 months (RR 1.07, CI 0.53–2.13, *p* = 0.87), 39% (23/59) if started between 6 and 11 months (RR 1.83, CI 0.97–3.46, *p* = 0.06), 47% (21/45) if started between 12 and 23 months (RR 2.19, CI 1.17–4.13, *p* = 0.01), and 70% (37/53) if started ≥ 24 months (RR 3.28, CI 1.74–5.84, *p* < 0.001) (Fig. 1).

**Fig. 2:**
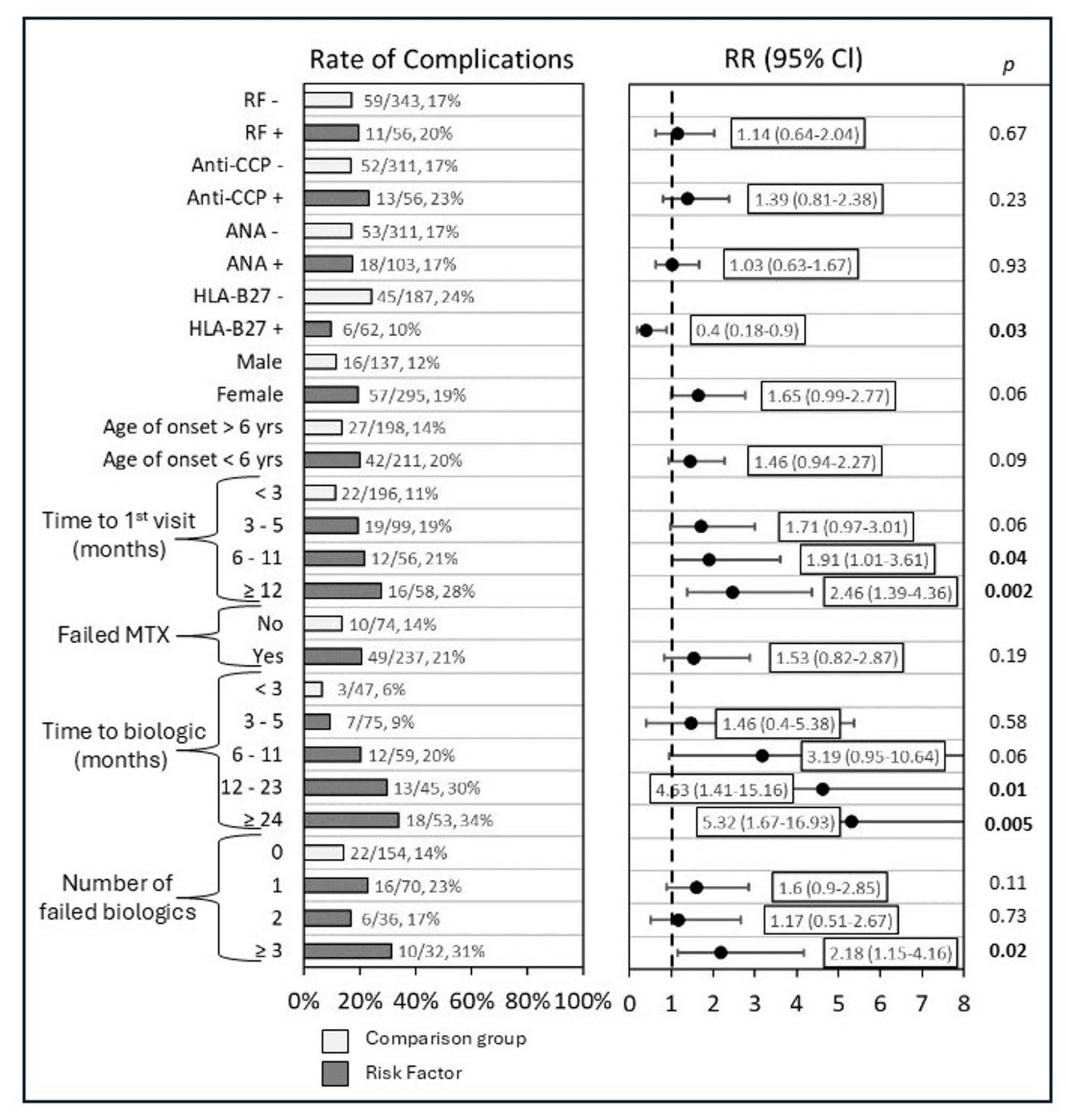
Risk Factors of developing joint complications. Shows proportion of patients of various factors who developed a joint complication. Relative risk with 95% confidence internal shown. Light bars represent comparison group, while dark bars show risk factors assessed for relative risk with 95% confidence internal as compared to the comparison group

Other factors assessed which did not have statistically significant increased risk of developing complications included serology positivity (RF, anti-CCP, ANA, HLA-B27), sex, time from disease onset until first rheumatology visit, failing methotrexate, and number of failed biologic and/or JAKi medications.

### Assessing risk factors of developing joint complications

Several factors were associated with a significant increased relative risk of developing joint complications, including increased time from disease onset to initial rheumatology visit, increased time to initiating biologic therapy, and failure of three or more biologic and/or JAKi medications (Fig. 2).

Increased time from disease onset until first rheumatology visit was found to have increased relative risk of developing a joint complication. Of patients who were seen within 3 months of disease onset, 11% (22/196) developed a joint complication. This compared to 19% (19/99) of patients who were seen between 3 and 5 months after disease onset (RR 1.70, CI 0.97–3.01, *p* = 0.06), 21% (12/56) who were seen between 6 and 11 months (RR 1.91, CI 1.01–3.61, *p* = 0.046), and 28% (16/58) who were seen ≥ 12 months after disease onset developed a joint complication (RR 2.46, CI 1.39–4.36, *p* = 0.002).

Time to biologic therapy initiation was also associated with increased relative risk to developing a joint complication. Specifically, 6% (3/47) of patients who began biologic therapy within 3 months of their first rheumatology visit developed a disease complication, compared to 9% (7/75) of patients starting between 3 and 5 months (RR 1.46, CI 0.4–5.38, *p* = 0.58), 20% (12/59) if started between 6 and 11 months (RR 3.19, CI 0.95–10.64, *p* = 0.06), 30% (13/45) if started between 12 and 23 months (RR 4.63, CI 1.41–15.16, *p* = 0.01), and 34% (18/53) if started ≥ 24 months (RR 5.32, CI 1.67–16.93, *p* = 0.005).

Of patients who did not fail a biologic and/or JAKi medication, 14% (22/154) developed a joint complication. Failing one biologic or JAKi (RR 1.6, CI 0.9–2.85, *p* = 0.11) or two biologic and/or JAKi medications (RR 1.17, CI 0.51–2.67, *p* = 0.73) did not have significantly increased relative risk. However, failing ≥ 3 biologic and/or JAKi medications was found to have significantly increased relative risk, with 31% (10/32) of patients developing a joint complication (RR 2.18, CI 1.15–4.16, *p* = 0.017).

Female sex showed a trend toward increased risk of developing joint complications, although this finding was not statistically significant (RR 1.65, CI 0.98-2,77, *p* = 0.06).

HLA-B27 positivity was associated with decreased relative risk of developing a joint complication, with 10% (6/62) of HLA-B27 positive patients developing a joint complication compared to 24% (45/187) of HLA-B27 negative patients (RR 0.40, CI 0.18–0.90, *p* = 0.026).

No statistically significant differences in relative risk were observed for other serologic markers including RF, anti-CCP, and ANA. Age of disease onset and failing MTX were also not found to have increased relative risk of developing joint complications.

### Assessing risk factors of developing uveitis

**Fig. 3:**
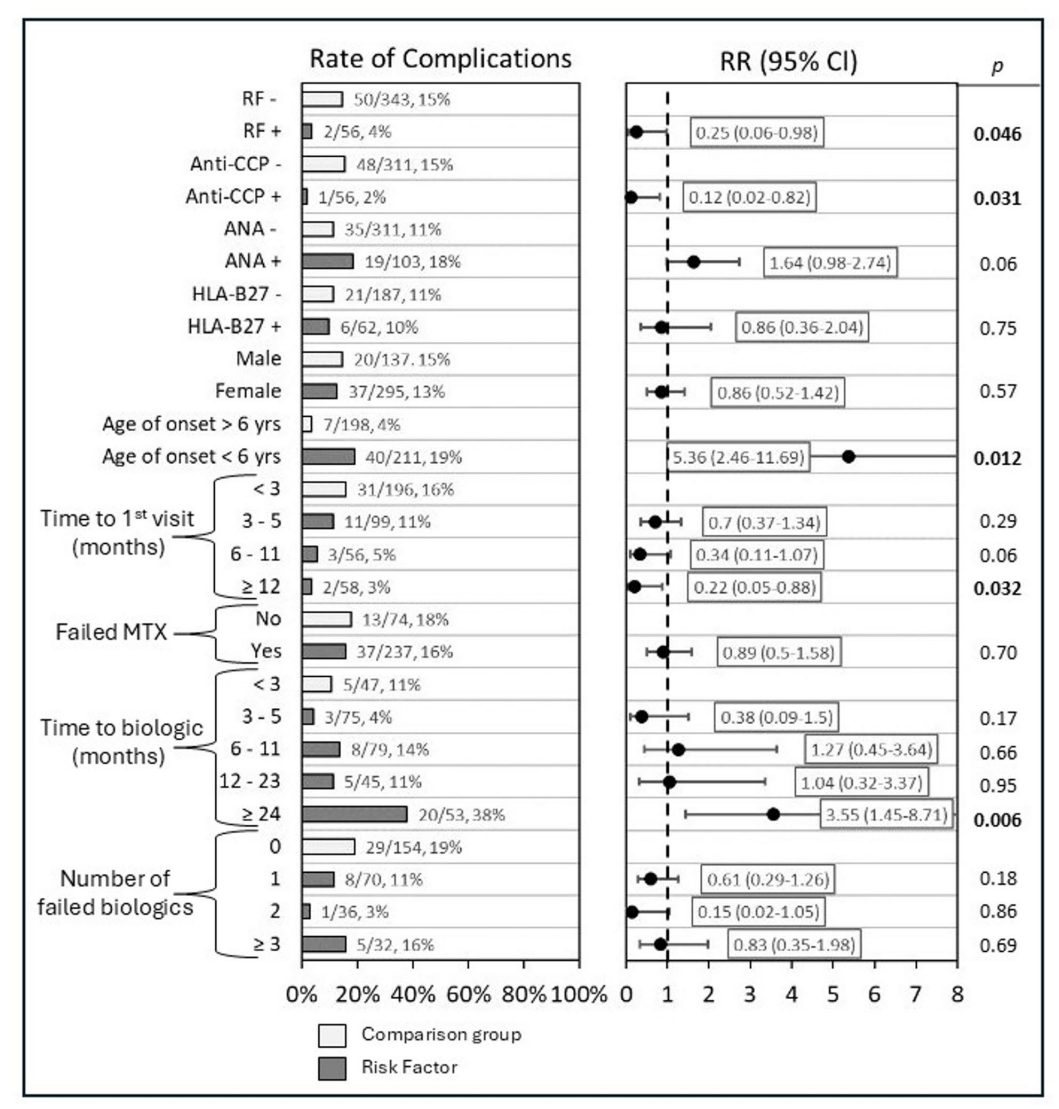
Risk Factors of developing uveitis. Shows proportion of patients of various factors who developed uveitis. Relative risk with 95% confidence internal shown. Light bars represent comparison group, while dark bars show risk factors assessed for relative risk with 95% confidence internal as compared to the comparison group

We also assessed potential risk factors for relative risk of developing uveitis. Age of disease onset < 6 years (RR 5.36, CI 2.46–11.69, *p* = 0.01) and starting a biologic medication after ≥ 24 months (RR 3.54, CI 1.45–8.71, *p* < 0.006) were found to have statistically significant increased relative risk of developing uveitis. ANA positivity trended towards increased risk, with 18% (19/103) of ANA positive patients compared to 11% (35/311) of ANA negative patients developing uveitis, however, this was not statistically significant (RR 1.64, CI 0.98–2.74, *p* = 0.06) (Fig. 3).

RF positivity (RR 0.25, CI 0.06–0.98, *p* = 0.046) and anti-CCP positivity (RR 0.12, CI 0.02–0.82, *p* = 0.03) were found to have decreased relative risk of developing uveitis.

No statistically significant associations were observed for HLA-B27 positivity, sex, time to first rheumatology visit, failing MTX, time until starting a biologic medication < 24 months, and number of failed biologic/JAKi medications.

## Discussion

Despite advances in treatment, approximately one-third of patients in our JIA cohort developed at least one disease complication, most commonly joint complications and uveitis. Complications were more frequent among patients with a younger age of disease onset, a longer duration of disease, and longer time to starting methotrexate and biologic therapies. However, these characteristics were found to be predominantly associated with uveitis and were not found to be associated with developing joint complications.

Oligoarticular JIA had the highest complication rate, primarily due to the high rates of uveitis in patients with oligoarticular disease. Conversely, disease subtypes such as ERA and RF positive polyarticular showed the lowest rates of developing disease complications. It is possible that the lower rates of complications in these subtypes may in part be explained by a shorter mean disease duration at time of assessment, as well as having low rates of uveitis.

One-sixth of patients developed joint complications, most commonly chronic arthropathy, which most frequently affected the fingers and wrists. Joint complications were identified most frequently in undifferentiated, RF negative polyarticular, and extended-oligoarticular JIA subtypes. However, the small sample size in our population with undifferentiated and extended-oligoarticular JIA subtypes may have inflated these rates.

Interestingly, we observed a lower-than-expected rate of chronic joint changes in patients with a RF positive polyarticular subtype. We did not observe an increased risk of joint complications with RF or anti-CCP positivity, despite prior studies linking these markers to greater joint damage [14–17]. One possible explanation is that RF positive polyarticular patients received more aggressive early treatment with methotrexate and/or biologic medication, which may have reduced joint damage. Additionally, higher mean age of disease onset and shorter disease duration in RF positive polyarticular patients may have contributed to the lower rate of joint complications identified in this subtype. It is possible that joint damage in this group may accumulate over time and become more apparent with longer follow-up into adulthood.

Risk factors significantly associated with joint complications in our JIA cohort included longer time from disease onset to first rheumatology evaluation, longer time to initiating biologic therapy, and failure of three or more biologic/JAKi agents. Specifically, delays of 6 months or more from symptom onset to initial rheumatology visit, and delays of 12 months or more in initiating biologic therapy, were associated with a significantly increased risk of joint complications. HLA-B27 positivity was associated with a lower risk of joint complications; however, this may reflect the lower complication rates observed in ERA, a subtype commonly associated with HLA-B27 positivity, rather than a direct protective effect.

Uveitis occurred in 13% of patients, consistent with previously reported rates ranging from 8 to 22%, depending on geographic and population differences [4, 18–21]. Oligoarticular-persistent JIA was the most commonly affected subtype, and younger age at onset was a strong predictor of uveitis, both findings consistent with prior literature [4–5, 20–21]. Patients with uveitis had a longer time to initiation of methotrexate and biologic therapy. Although ANA positivity is a well-established risk factor for uveitis [5, 18, 20–21], our study did not reach a statistical significance for this association (*p* = 0.06), likely due to sample size limitations. In contrast, both RF and anti-CCP positivity were found to correlate with lower rates of uveitis as previously reported [20].

Secondary ocular complications developed in 25% of patients with uveitis including cataracts and glaucoma. Patients with secondary ocular complications had longer disease duration compared to patients with uveitis without secondary complications, however, no other differences or predictive factors were observed between these groups. Given our small sample size of patients with secondary ocular complications, it is possible that predictive factors of developing secondary ocular complications were not identified which may have been revealed with a larger population.

Growth failure was observed in 4% of patients, with short stature resulting in 1%. These rates are lower than previously reported figures, which range from 10 to 40% [22–25]. Among patients with systemic JIA, 14% had growth failure and 5% developed short stature - lower than historical rates [22–24]. Differences in definitions of growth failure may explain these discrepancies [22, 23, 25]. We used a more stringent definition (a decrease in height z-score > 1.0 and short stature defined as a final z-score < − 2.0) to measure severe growth failure, which may partially explain our lower reported rates. Our study did not account for parental height, which may have limited the precision of short stature assessment. Additionally, because baseline height data prior to rheumatology care were unavailable, some early growth restriction may have gone unrecorded. It is also possible that catch-up growth following treatment initiation may have mitigated observed height deficits by the time of final measurement.

Among patients with systemic JIA, 20% developed MAS, a rate consistent with findings from a multinational cohort study reporting 22% [8], and somewhat higher than earlier estimates of 10% [26, 27].

In our assessment, one of the more prominent risk factors for disease complications identified included time to initiation of biologic therapy. Nearly 70% of patients who started biologic treatment two or more years after their initial visit developed a disease complication. Early initiation of biologic therapy appears to have a protective effect against developing joint complications, with only 6% of patients developing a joint complication who were started on a biologic medication within three months of their first rheumatology visit. The risk of developing joint complications increased with prolonged time to initiation of biologic therapy, such that delaying biologic treatment until after six months increased risk by three-fold. The risk of joint complications increased to more than four-and-a-half times if starting a biologic after 12 months, and to more than five times if starting after 24 months. This demonstrates the importance of early initiation of biologic medications in preventing the development of disease complications.

Limitations of this study included being conducted at a single center and having a short study period. As only patients actively being followed during the study period were included, patients diagnosed with JIA in recent years but who were lost to follow up prior to the study period were not included in the study. Thus, a disproportionately higher number of patients with disease remission may have been excluded, which may have falsely inflated the rates of complications reported. Furthermore, this report is limited to the clinical experience of disease complications developing in childhood and does not assess the rates of complications seen into adulthood.

## Conclusions

In conclusion, this study is the first to our knowledge to provide a comprehensive assessment of disease complication rates in a contemporary cohort of patients with JIA using a prospective study. Despite therapeutic advances in the treatment of JIA, a substantial proportion of patients continue to develop disease complications. Delayed initiation of biologic therapy was associated with higher complication rates, highlighting the importance of early therapeutic intervention to reduce the burden of disease complications. Further studies will be needed to reaffirm these findings, particularly in larger, multi-site populations. Studies tracking patients into adulthood would also allow for a more robust description of long-term complications.

## Data Availability

The datasets used and/or analyzed during the current study are available from the corresponding author on reasonable request.

## Abbreviations

AJC: Active joint count
ANA: Antinuclear antibodies
cJADAS: Clinical juvenile arthritis disease activity score
CI: Confidence Intervals
CDC: Control and Prevention
CCP: Cyclic citrullinated peptide
DMARD: Disease-modifying antirheumatic drugs
ERA: Enthesitis-related arthritis
HLA-B27: Human leukocyte antigen B27
IRB: Institutional Review Board
ILAR: International League of Associations for Rheumatology
JAKi: Janus kinase inhibitors
JIA: Juvenile Idiopathic Arthritis
MAS: Macrophage activation syndrome
MTX: Methotrexate
NSAIDS: Nonsteroidal anti-inflammatory drugs
RR: Relative risks
RF: Rheumatoid factor
TNFi: Tumor necrosis factor inhibitors
WHO: World Health Organization
